# A standardized suprapubic bottom-to-up approach in robotic right colectomy: technical and oncological advances for complete mesocolic excision (CME)

**DOI:** 10.1186/s12893-019-0544-2

**Published:** 2019-07-01

**Authors:** Jan Schulte am Esch, Sergio-I. Iosivan, Fabian Steinfurth, Ammar Mahdi, Christine Förster, Ludwig Wilkens, Alaa Nasser, Hülya Sarikaya, Tahar Benhidjeb, Martin Krüger

**Affiliations:** 1 Center of Visceral Medicine, Department of General and Visceral Surgery, Center of Visceral Medicine, Protestant Hospital of Bethel Foundation, Schildescher Str. 99, Bielefeld, Germany; 2 Institute of Pathology, KRH Clinic Nordstadt, Hannover, Germany; 3 Center of Visceral Medicine, Department of Internal Medicine and Gastroenterology, Protestant Hospital of Bethel Foundation, Bielefeld, Germany

**Keywords:** Colon carcinoma, Complete mesocolic excision, Laparoscopy, Right colectomy, Robotic surgery, Lymph nodes

## Abstract

**Backround:**

Several studies have demonstrated a direct correlation between lymph node yield and survival after colectomy for cancer. Complete mesocolic excision (CME) in right colectomy (RC) reduces local recurrence but is technically demanding. Here we report our early single center experience with robotic right colectomy comparing our standardized bottom-to-up (BTU) approach of robotic RC with CME and central vessel ligation (CVL) facilitated by a suprapubic access with the “classical” medial-to-lateral (MTL) strategy.

**Methods:**

A 4-step BTU approach of robotic RC guided by embryonal planes in the process of retrocolic mobilization with suprapubic port placement was performed in the BTU-group (*n* = 24; all with intention to treat cancer). In step 1 CME was initiated with caudolateral mobilization of the right colon guided by the fascia of Toldt across the duodenum and up to the Trunk of Henle. Subsequently, dissection was performed BTU right of the middle supramesenteric vessels with central ileocolic vessel ligation in step 2. Subsequent to separation of the transverse retromesenteric space and completion of mobilization the hepatic flexure in step 3, the transverse mesocolon was then transected right of the middle colic vessels in step 4. An extracorporeal side to side anastomosis was performed. We compared the outcome of the BTU-group with a MTL-group (*n* = 7).

**Results:**

Patient characteristics like age, gender, BMI, comorbidity (ASA) and M-status were comparable among groups. There was no conversion. Overall complication rate was 35.5%. We experienced no anastomoses insufficiency, grade Dindo/Clavien III/IV complication or mortality in this study. Type I and II complications and surgical characteristics incl. OR-time, ICU- and hospital-stay were comparable between the two groups. However, the lymph node yield was superior in the BTU-group (mean 40.2 ± 17.1) when compared with the MTL-group (16,3 nodes ±8.5; *p* <  0,001).

**Conclusions:**

Compared to the classical MTL approach, robotic suprapubic BTU RC changes from a search of the layers bordering the oncological dissection to a consequent utilization of the planes as a retro-mesocolic guide during CME. The BTU strategy could bear the potential to increase the lymph node yield. Robotic systems may provide the technical requirements to combine advantages of both open and minimally invasive RC.

## Backround

Minimally invasive resection concepts for right colectomy (RC) must fulfil the same oncologic criteria as compared with an open approach which includes “no-touch isolation technique”, ligation of the vascular pedicles at their origin, oncologic lymphadenectomy and “distal and radial clearance” of the neoplasm from resection margins [1].

Although still inconclusive as based predominantely on retrospective studies [2] en bloc resection of the lymphatic drainage of the malignant neoplasia, comprising associated lymph nodes performing complete mesocolic excision (CME) in combination with central vessel ligation (CVL) has been repetitively demonstrated to bear the potential of improving overall and disease specific survival following surgery for CRC [3–7].

Three key aspects for CME plus CVL found a consensus as a gold standard in RC: development and separation of the right retrocolic connecting fascia to develop an unhurt mesocolon as a sound cluster, CVL with dissection of the ileocolic vessels at their origin to optimize the vertical lymph node dissection for regional control and achievement of an appropriate length of colon to remove pericolic lymph nodes, maximizing the collection of longitudinal lymph nodes [8]. However, in contrast to total mesorectal excision (TME) for rectal cancer, routine implementation of CME in RC has yet not been achieved to a comparable level [9].

**Fig. 1:**
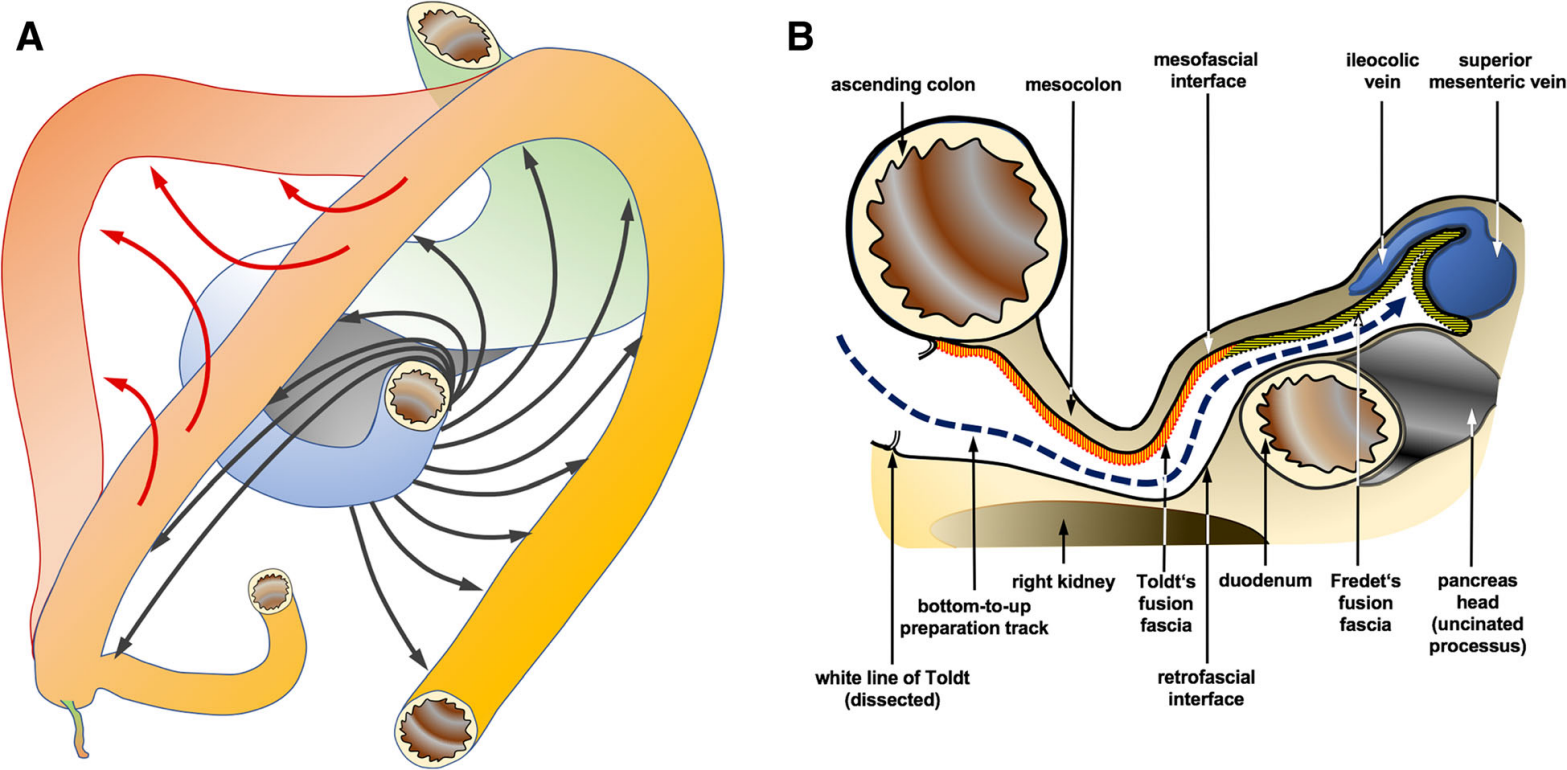
**a** Embryological rotation and folding of the colon (drawing art by author J. Schulte am Esch). **b** Layers and structures relevant for down-to-up dissection of the retro-mesocolic space in robotic right-colectomy (drawing art by author J. Schulte am Esch)

This is despite the demonstrated oncological improvement and the principles underlying CME being anatomically logical. The complex embryonic rotation around the mesenteric root and subsequent folding (Fig. 1a) implicates a set of planes that comprises layers and surfaces relevant for RC. In this context, critical planes for retrocolic dissection are the fascia of Toldt (right retrocolic fascia) and in upper medial continuity with Fredet’s fascia (fascia preduodenopancreatica) up to the superior mesenteric vein (SMV). These fusion structures are ventrally delineated by the posterior layer of the ascending mesocolon (the meso-fascial interface) and dorso-laterally, by the prerenal fascia, representing the posterior parietal peritoneum covering the retroperitoneum (the retro-fascial interface) [8]. Laterally the white line of Toldt marks the access to Toldt’ fascia (Fig. 1b).

Lately, first reports of the concepts of robotic right colectomy (RRC) incorporating CME and CVL came up mainly following the adaption or modification of the medial to lateral approach as established in laparoscopic techniques [10–13]. In a first study reported by Petz and colleagues a suprapubic approach was utilized [14]. Here we propose a standardized four-step procedure of RRC implementing suprapubic port positioning as introduced lately [15] with a consequent down-to-up approach latter with improved implementation of CME and CVL.

## Methods

### Study design and robotic system

We conducted this retrospective single center study on 31 consecutive patients with a robotic hemicolectomy with approval by concerned local ethics committee. The included patients in this study were operated at our center from July 2016 to August 2018 with the intention to treat oncologically a right sided colon cancer. Surgery was performed with the DaVinci Xi® system (Intuitive Surgical, Aubonne, Switzerland) which is connected to a TruSystem® 7000dV OR-table (TRUMPF Medizin Systeme, Saalfeld, Germany) enabling integrated table motion without necessity of detaching the robotic device.

### System setup and study groups

**Fig. 2:**
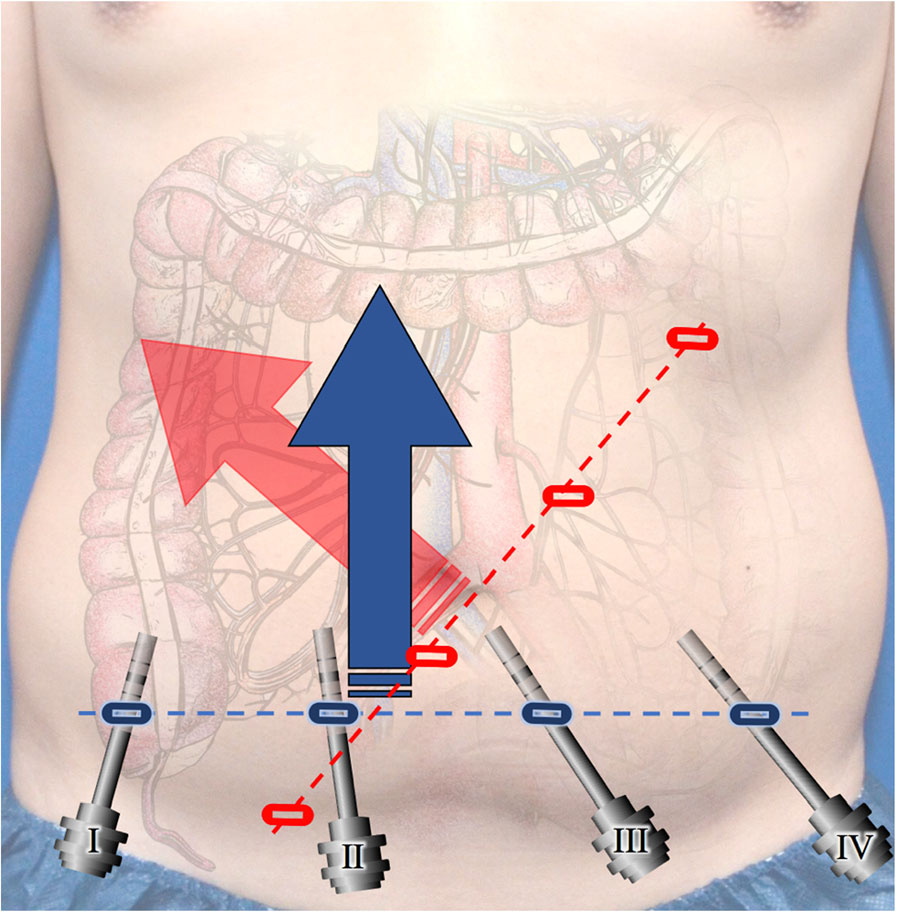
Port positioning in RRC with suprapubic bottom-to-up (blue) vs. classical medial to lateral approach (red). Arrows indicate main surgical orientation of each approach (drawing art by author J. Schulte am Esch)

Instead of a traditional angled line running from lower medial to left upper abdomen we used the suprapubic robotic trocar setup, positioning the 4 ports along a horizontal line 3–5 cm above the pubis (Fig. 2) as introduced lately by Yeo et al. [15] plus 1 OR-table assistant operated 13 mm-trocar in the left lateral abdomen. Pneumoperitoneum was set to a pressure of 11 to 12 mmHG. All patients were positioned in a 23–25° head down and 12 to 14° left sided orientation of the OR-table that provides an optimal position for retro-colic dissection as well as superior-mesenteric vessel-development with CVL. Patients in the BTU-group (*n* = 24) received surgery with the newly adopted strategy of RRC with a bottom to up strategy whereas individuals in the MTL-group (*n* = 7) were operated with classical medial to lateral approach. Both procedures have been performed by the same surgeon during the same time period.

### Surgical technique in the BTU-group

**Fig. 3:**
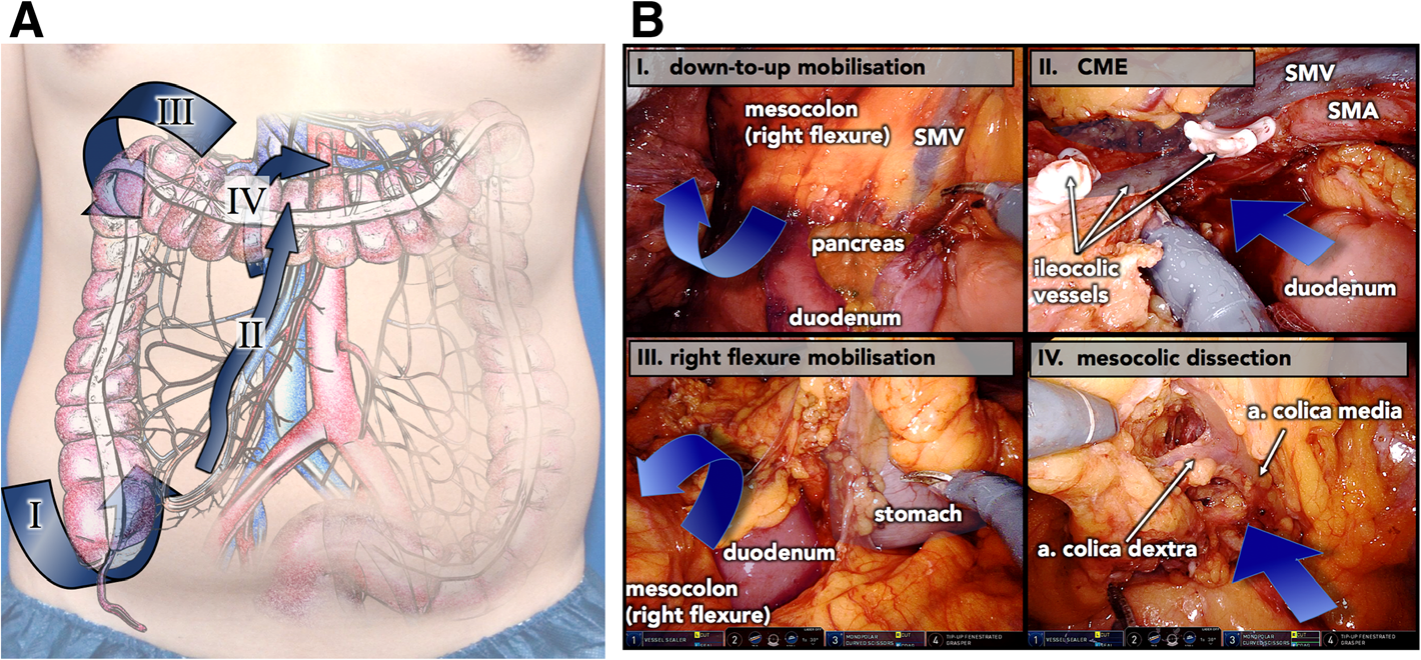
**a** Visualisation of the 4 key steps in robotic right colectomy with suprapubic approach, CME and CVL with respect to anatomical positioning (drawing art by author J. Schulte am Esch). **b** Representative intra-operative still photographies of the 4 key steps in robotic right colectomy with suprapubic approach, complete mesocolic excision (CME) and central vessel ligation. SMV – superior mesenteric vein; SMA – superior mesenteric artery

Patients in the BTU-group underwent the new 4-step concept of bottom-to-up mobilization and embryonic layers guided dissection of the retrocecal, ascending and transverse mesocolon followed by completion of CME and specimen development via transection right along superior mesenteric vessels and CVL next to their junction (Fig. 3a+b). Step 1 of the procedure, the caudo-lateral mobilization of the right colon, was initiated with cleavage of the lateral “white line of Toldt” around the cecum, along the ascending colon and around the hepatic flexure. Dissection continued between the retro-fascial and the meso-fascial interface. Consequently, a bottom-to-up oriented detachment retro-mesocolically along the fascia of Toldt and ventral to the duodenum and the Pancreas head along Fredet’s fascia exposing all essential planes for CME and CVL of ileocolic vessels *prior* to dissection along superior mesenteric vessels and high branches of the trunk of Henley. Mobilizing the duodenum, as suggested by Hohenberger et al. [3] was avoided in all cases, leaving the Treitz fascia (fascia retropancreatica) intact. The initial complete mobilization of the retro-mesocolic space in step 1 eases the identification of the superior mesenteric vessels and their ramification throughout the following steps.

The down-to-up orientation consequently provided by the suprapubic port positioning facilitates control of the specimen also from the dorsal aspect in the process of dissection in step 2 performed consistently along the right lateral aspect of superior mesenteric vessels and their inconsistent branches. Following transection of the ilecocolic vessels right next to their origin (CVL), en-bloc lymphadenectomy of the anterior aspect of the SMV from the ileocolic vessel stump towards the base of the middle colic artery was completed. In step 3 transection of the transverse retro-mesenteric space and mobilization of the hepatic flexure were completed prior to the transection of the transverse mesocolon in step 4 right of the middle colic vessels. Last associations to the mesenteric root were transected achieving a complete dissected specimen just attached by the bowel.

### Surgical technique in the MTL-group

Before switching to the BTU-strategy classical medial to lateral development of the CME-envelope was performed in the initial phase of this series on patients that are here summarized as the MTL-group. Following dissection of the specimen right lateral of the superior mesenteric vein down to the fascia of Treitz the dorsal aspect was developed towards the white line of Treitz. Following the dissection of Treitz faszia, Fredet’s Fascia ventral the duodenum and pancreas head was dissected towards the mesenteric root. Mobilisation of the resection specimen was completed similar to steps 3 and 4 in the BTU-group in a medial to lateral orientation of the preparation path.

### Specimen retrieval and anastomosis

Specimen retrieval and anastomotic technique were identical in both groups. With a robotic clamp, the determined position of transverse colon dissection was pointed out to the OR-table side assistant from the peritoneal side of the abdominal wall. That mark indicated the medial margin of the horizontal mini-laparotomy in the left upper quadrant being extended 5 to 7 cm to the left, serving as an access for specimen retrieval and to perform the anastomosis. This access to the abdomen was achieved by cutting the superficial and deep layer of the abdominal rectus muscle fascia subsequent to cutaneous and subcutaneous cut, separating the vertically divided muscle fibers to the sides with a circular foil retractor without muscular disruption. Following externalization of the specimen starting from the terminal ileum, transverse colon and ileum were dissected with a linear cutter at the position of adequate perfusion, determined by visualization complemented in the last 15 cases with a fluorescence angiography utilizing 15 mg indocyanine green. Ileo-transversostomy was performed in all cases side-to-side extracorporeally with a combination of linear stapler and buried by a running suture on top.

### Statistics

Statistical analysis was performed with SYSTAT© 13.1 (Systat Software, Inc.,San Jose, CA, USA) and Stata© 15.1 (StataCorp, College Station, TX, USA) software. With respect to the small number of individuals in the MTL-group we selected statistical methodology conservatively. For continuous variables, assuming non-normal distribution, two-sample Wilcoxon rank-sum (Mann-Whitney) test was utilized to compare the two groups. To give credit to the small sized MTL-group Fisher’s exact test was performed on categorical variables. A *p*-value of < 0.05 was regarded to be significant.

## Results

### Pre-operativ patients basic characteristics

Mean age of patients in the BTU- and the MTL-group was 72.5 ± 7,9 years and 78.1 ± 8.8 years respectively. Gender, BMI and co-morbidity indicating ASA score were comparable between both groups (Table 1). All patients in this study were free of distant metastases (M0) in pre-surgical staging assessed by computed tomography-scan of chest and abdomen as well as in ultrasound examination of the liver with contrast medium.

**Table 1:** Pre-operativ patients’ basic characteristics

	bottom-to-up (BTU-group) *n* = 24	medial-to-lateral (MLT-group) *n* = 7	*p*-value
Age, years (mean ± SD)	72.5 ± 7,9	78.1 ± 8.8	0.156 (W)
Sex (% (n))			
Male	29,2 (7)	28,6 (2)	0.976 (F)
Female	70,8 (17)	71,4 (5)	
BMI (mean ± SD)	24.7 ± 3.6	28.0 ± 4.4	0.156 (W)
ASA score (% (n))			
1	0.0 (0)	12.5 (3)	0.849 (F)
2	42.9 (3)	45.8 (11)	
3	57.1 (4)	41.7 (10)	
M0-Stage (% (n))	100 (17)	100 (7)	1.000 (F)

*n* Numbers in brackets, *W* Two-sample Wilcoxon rank-sum (Mann-Whitney) test, *F* Fisher’s exact test, *SD* Standard deviation

### Oncological outcome

Seventeen patients in the BTU-group (68%) were diagnosed with colon cancer in the final histopathology report following resection. The remaining patients in this group revealed an adenoma with high grade dysplasia. All 7 patients in the MTL-group were positive for colon cancer on final histopathology assessment. Oncological parameters in cancer patients including TNM-stage and number of positive lymph nodes were comparable between both groups (Table 2). Two out the 7 patients in the MTL-group (28.6%) were below the critical total number of lymph nodes (< 12) for adequate T-staging [16] whereas none of the cases in the BTU-group revealed a lymph node count below 12 in all cases evaluated by the acetone compression method. This difference between both groups was statistically significant (*p* = 0.045). Accordingly, the mean lymph node yield of 16.3 ± 8.5 was significantly lower in the MTL-group when compared to the BTU-group with a mean of 40.2 ± 17.1 nodes (*p* <  0.001).

**Table 2:** Oncological and surgical outcome

	bottom-to-up (BTU-group)	medial-to-lateral (MLT-group)	*p*-value
	*n* = 17	*n* = 7	
T-stage (%(n))			
1	11,8 (2)	14.3 (1)	0.153 (F)
2	11,8 (2)	28,6 (2)	
3	70,6 (12)	28,6 (2)	
4	5,9 (1)	28,6 (2)	
N-Stage (%(n))			
0	58,8 (10)	85,7 (6)	0.332 (F)
1	29,4 (5)	0.0 (0)	
2	11,8 (2)	14,3 (1)	
Tumor involved margins (%(n))	0 (0)	0 (0)	1
Total no. retrieved lymph nodes			
mean ± SD	40.2 ± 17.1	16.3 ± 8.5	< 0.001 (W)
median (range)	38 (14–86)	12 (9–30)	
Cases with fewer than 12 LNs (%(n))	0.0 (0)	28.6 (2)	0.045 (F)
No. of positive lymph nodes, median (range)	0 (0–15)	0 (0–5)	0.272 (W)
	*n* = 24	*N* = 7	
No. of conversions	0 (0)	0 (0)	1.000 (F)
Skin-to-skin OR-time (min; mean ± SD)	283.6 ± 87.9	287.5 ± 45.0	0.671 (W)
time to first flatus (d; mean ± SD)	1.4 ± 1.1	0.6 ± 1.0	0.074 (W)
ICU stay (d; median (range))	0.0 (0–8)	0.0 (0–1)	0.534 (W)
Hospital stay (d; mean ± SD))	10.7 ± 2.6	11.6 ± 3.7	0.721 (W)
Patients with complications (%(n))	33.3 (8)	42.9 (3)	0.676 (F)
Patients with DinCla complications (%(n))			
DinCla I complications	29.2 (7)	28.6 (2)	0.633 (F)
DinCla II complications	4.2 (1)	14.3 (1)	
DinCla III/IV complications	0.0 (0)	0.0 (0)	
Postoperative mortality / DinCla V (%(n))	0.0 (0)	0.0 (0)	1.000 (F)
Type of morbidity (%(n))			
Surgical site infection	8.3 (2)	0.0 (0)	1.000 (F)
Ileus	0.0 (0)	0.0 (0)	1.000 (F)
Anastomotic leakage	0.0 (0)	0.0 (0)	1.000 (F)
anemia	4.4 (1)	14.3 (1)	0.406 (F)
Pneumonia	12.5 (3)	0.0 (0)	1.000 (F)
urinary tract infection	4.4 (1)	0.0 (0)	1.000 (F)
lymphatic fistula	4.4 (1)	0.0 (0)	1.000 (F)
incisional hernia	4.4 (1)	14.3 (1)	0.406 (F)

DinCla – According to Dindo/Clavien classification of complications [17]. *W* Two-sample Wilcoxon rank-sum (Mann-Whitney) test, *F* Fisher’s exact test, *SD* Standard deviation, *A* ANOVA. *P*-values of < 0.05 were regarded to be significant

### Surgical outcome

We experienced no conversion to classic laparoscopic or open surgery in this study (Table 2). Mean operating time (skin-to-skin) was 283.6 ± 87.9 min in the BTU-group and 287.5 ± 45.0 min in the MTL-group (*p* = 0. 671). Mean time to first flatus was 1.4 ± 1.1d vs. 0.6 ± 1.0d demonstrating a trend of a delay in the BTU-group (*p* = 0.074). There were no statistical differences between BTU-group and MTL-group with regard to length of ICU (median (range): 0.0d (0–8) vs. 0.0 (0–1); *p* = 0.534) and hospital-stay (mean ± SD: 10.7 ± 2.6 vs. 11.6 ± 3.7; *p* = 0.721).

Overall 35.5% of the patients included in this study experienced complications (all grade I/II according to Dindo/Clavien [17]). There was no anastomoses insufficiency, grade Dindo/Clavien III/IV complication or mortality observed in this study. In the BTU-group in 29.2% (*n* = 7) vs. the MTL-group in 28.6% (*n* = 2) of the patients we observed one or more postoperative type I complication and in 4.2% (*n* = 1) vs 14.3% (*n* = 1) type II complications respectively according to Dindo/Clavien. Those included one case each group of anemia requiring blood transfusion as type II complication. In the BTU two surgical site infections (both superficial wound infections in the BTU-group), one case of urinary tract infection and one lymph fistula latter quickly responding to fatty diet adjustment (medium chained fatty acids) were observed. The total number of patients with type I and type II complications as well as the specific kind of complications were not statistically different among the two groups (Table 1). As late (> 30d postoperative) complications one trocar-incisional hernia was observed in each group.

## Discussion

In our experience, the novel suprapubic access in combination with the down-to-up approach facilitates technically improved CME and CVL in robotic right colectomy if compared to the “classical” MTL strategy. The number of retrieved lymph nodes was significantly increased by the here presented BTU-approach of RRC when compared with MTL. Although operating on rather elderly patients, we demonstrated the procedure in this early experience to be safe observing moderate morbidity comparable between both groups.

Recently, RRC has been praised as an alternative to classic laparoscopic RC [12, 18]. CME allows for resection along the unimpaired contour delineating the dorsal mesentery and the anatomo-embryological fusion fascias and is therefore vital for a true radical R0 resection, as the mesocolon contains the whole potential routes for local neoplastic spread through lympho-vascular, neuro-perineural and fibro-fatty tissues [3, 9, 19–21]. CVL facilitates a comprehensive lymph node harvest along the supplying vessels paralleled by a relevant effect on regional recurrence and systemic dissemination [8, 22]. Lateral access is applied for open CME, during which the right colon is mobilized lateral to medial firstly along Toldt’s fascia; then, the visceral fascia is sharply dissected right sided along superior mesenteric vessels in order to expose and dissect the right colon-feeding vessels. In contrast, the laparoscopic CME to date applies a medial approach, during which the visceral peritoneum along the root of the right mesocolon is cut and lymph nodes lining up the surgical trunk are dissected firstly, followed by the seek and separation of Toldt fascia and ligation of the central vessels. Latter technique was applied here in the MTL-group.

Several laparoscopic concepts were presented to adapt the anatomical defaults of an oncological RC primarily proposed for open RC [3, 23]. Feng et al. proposed a hybrid medial approach of laparoscopic right colectomy prospectively compared to a completely medial approach. The hybrid approach is based on a first up-to-down dissection (separation of the gastrocolic ligament and dissection of the middle colic vessels and Henle’s trunk) combined with a subsequent classical medial-to lateral down-to-top approach. The complete medial approach on the other hand, involved a down-to-up approach in every step, including the dissection of the middle colic vessels and the Henle trunk as well as dissection of the inferior edge of the pancreas. Like in all medial to lateral access variants, authors pointed out that in any case, surgical and oncological safety are critically depending on the precise initial identification of the anatomical planes after dissection of the medial and transverse mesocolic peritoneum [24]. Matsuda et al. also implemented a variation of a cranio-to-caudal approach in their concept of laparoscopic right meso-colectomy, noting that lymph node dissection around the middle colic vessels is technically demanding. Authors stressed the necessity of detailed knowledge of the embryonic developed structures and planes to dissect the retro-mesocolic space [25, 26]. These and other reports illustrate the challenge of minimally invasive RC implying CME and CVL to meet both patient and oncological safety demanding meticulous knowledge of the anatomy to unveil the correct planes.

When compared with the classical laparoscopic medial to lateral approach the here proposed robotic concept may adopt the requirements for oncological optimization in RC by switching from a search of the planes (Toldt’s / Fredet’s fascia) towards a consequent utilization of the planes as dissection guide from the beginning (white line of Toldt, Fig. 1a) prior to any vessel ligation keeping the “envelope” of the resection specimen intact. In addition, initial mobilization in combination with a head down angulation of patient positioning (Trendelenburg) facilitates CME in caudal parts, that otherwise would be difficult to reach with the suprapubic port positioning. Furthermore, the consequent bottom-to-up approach, permits subsequent dissection of the retro- mesocolic space along the fascia of Toldt and ventral to the duodenum as well as the pancreas head exposing all the essential planes for CME and CVL prior to vessel dissection and ligation. The initial complete mobilization of the mesocolon eases the identification of the superior mesenteric vessels. The suprapubic approach facilitates the dissection right lateral to the superior mesenteric vessels and along a dorsally mobilized and subsequently controllable mesocolon under optimized vision enabling the identification and dissection of the branches to the right colon in all their variations. Excessive tension on the ileocolic vessels is avoided by obtaining exposure through a local step-by-step traction-counter-traction strategy. This may reduce the known risk factors of iatrogenic vessel injuries in CME and CVL [27] and possibly simplifies the procedure especially in obese patients who represent a challenge with conditions including missing the space for tension on ileocolic vessels to adequately expose the medial plane of the ascending mesocolon in the classical medial to lateral approach. In addition, the true bottom-to-up alignment of the procedure allows continuous visual orientation during ligation of vessels to the right of the superior mesenteric vessels in the course of CME by deploying the mesenteric root and middle colic artery as targeting structures.

The mean lymph node yield of 16.3 in our MTL group of patients is comparable to those observed by other authors for classic laparoscopic right colectomy [1] and robotic assisted right colectomy [28, 29] if performed with MTL strategy. The mean lymph node retrieval in the BTU-cohort of 40.2 is superior to the range of levels reported for classic laparoscopic as well as for open techniques. The improved lymph node yield in robotic RC if compared to historic laparoscopic and open techniques is in accordance with other reports [29, 30]. Our levels of lymph node harvest were comparable to very recent early series of BTU-RRC [31]. We were able to reduce the number of procedures with lymph node harvest below the critical number of 12 [17] from 29% to zero with the BTU-approach. In light of improved technical approach and oncological achievements represented by increased lymph node yield we demonstrated an improvement with the bottom-to-up strategy in RRC over the medial to lateral path which may be even superior to open and laparoscopic strategies.

Operating time seems rather high in this series; however, it is comparable to other studies on RRC [32–34]. Mean skin-to-skin time is explainable by the early experience with this novel procedure. It is in the range of other reports on early stages of adopting techniques in minimally invasive techniques in RC [25, 33]. We operated in elderly patients with ASA scores manly ranging at 2 to 3 that decided us to prolong the hospital observation time of our patients leading to a comparatively mean length of hospital stay. The observations of other authors, comparing young and elderly patients with robotic colorectal surgery in general [35] plus our increasing experience with the procedure should shorten hospital stay over time.

This study has some limitation particularly concerning comparison of the effectiveness of lymph node harvest. For one, it is limited by the small number of patients especially in the MTL group. Consequentely statistical analyses on lymph node numbers between the two groups, although performed by conservative testing with respect to small groups size, has to be interpreted with caution. Further the fact, that all patients but one in the MTL group were operated in the initial phase of our robotic colorectal surgery program, may play a role for the lymph node count distinctions. Although differences were pronounced concerning lymph node harvest and the fact that other variables like operating time, hospital stay and complication profiles were comparable between groups, one can not exclude a bias for lymph node yield in favour of BTU-operated patients based on the learning curve.

## Conclusions

With the suprapubic approach, the utilization of robotic systems may not just target a simplification of the minimally invasive procedure of RC [1]. The here proposed standardized robotic four-step suprapubic approach with down-to-up mesocolic mobilization and subsequent CME plus CVL demonstrated to be safe even in elderly patients. It may bear the potential of exceeding a minimally invasive technique of RC from the stage of “being easier than laparoscopy” to an oncological advanced concept. Robotic systems as used by us and other groups may provide the technical requirements to combine advantages of both open and minimally invasive surgical concept in oncologic RC. The preliminary clinical results in this study need to be proven in a multi-center randomized setting in larger cohorts and on a long-term basis.

## Data Availability

The datasets used and/or analysed during the current study are available from the corresponding author on reasonable request.

## Abbreviations

ASA: American Society of Anesthesiologists
CME: Complete mesocolic excision
CVL: Central vessel ligation
ICU: Intensive care unit
OP: Operation
RC: Right colectomy
RRC: Robotic right colectomy
SD: Standard deviation
SMV: Superior mesenteric vein
